# Translation and cultural adaptation to the Brazilian context of the Organizational Readiness to Change Assessment scale

**DOI:** 10.1590/S2237-96222025v34e20240921.en

**Published:** 2026-03-09

**Authors:** Tânia R. Bertoldo Benedetti, Thaís Fávero Alves, Natalia Santos, Cassiano Ricardo Rech, Gaia Salvador Claumann, Fabiana Brito Silva

**Affiliations:** 1 Universidade Federal de Santa Catarina, Departamento de Educação Física, Florianópolis, SC, Brazil; 2 University of Nebraska Medical Center, College of Public Health, Omaha, NE, United States

**Keywords:** Evaluation of Research Programs and Tools, Cross-Cultural Comparison, Health Care Evaluation Mechanisms, Health Services Research, Validation Studies, Evaluación de Programas e Instrumentos de Investigación, Comparación Transcultural, Mecanismos de Evaluación de la Atención de Salud, Investigación sobre Servicios de Salud, Estudio

## Abstract

**Objectives:**

To translate and culturally adapt the Organizational Readiness to Change Assessment scale to the Brazilian context.

**Methods:**

The translation and cross-cultural adaptation process took place in six stages: 1) authorization from the author of the original scale; 2) two translations of the scale into Brazilian Portuguese and synthesis into a single version; 3) back-translation; 4) evaluation of the initial version by a review committee (ten participants): clarity, comprehension, and suitability to the Brazilian context; 5) expert assessment I (same participants as the previous stage): evaluation of semantic, idiomatic, cultural, and conceptual equivalences; 6) expert assessment II (seven experts from five Brazilian regions): evaluation of semantic, idiomatic, cultural, and conceptual equivalences, considering linguistic variations in Brazil, and establishment of the final version. The experts involved in the different stages were professionals with academic experience or working in public health. For the assessments in stages 5 and 6, the content validity index was applied, considering agreement of at least 80.0% among experts as acceptable.

**Results:**

The 20 questions of the scale and their additional items (a total of 74 items to be answered) were translated and adapted according to the needs identified at different stages of the process. In stages 5 and 6, the questions of the three domains of the scale (evidence, context, and facilitation) demonstrated adequate content validity. Two questions presented a content validity index <80.0% in some equivalences. These and other items for which modifications were suggested were adjusted, resulting in the final version of the scale.

**Conclusion:**

The scale was translated and culturally adapted to the Brazilian context, demonstrating adequate content validity.

## Resumen

**Objetivo:**

Traducir y adaptar transculturalmente, para el contexto brasileño, la escala *Organizational Readiness to Change Assessment*.

**Métodos:**

El proceso de traducción y de adaptación transcultural se realizó en seis etapas: 1) autorización del autor de la escala original; 2) dos traducciones de la escala al portugués y síntesis en una sola versión; 3) retrotraducción; 4) comité de evaluación de la versión inicial (diez participantes): claridad, comprensión y adecuación al contexto brasileño; 5) evaluación de especialistas I (los mismos de la etapa anterior): evaluación de las equivalencias semántica, idiomática, cultural y conceptual; 6) evaluación de especialistas II (siete especialistas: cinco regiones de Brasil): evaluación de las equivalencias semántica, idiomática, cultural y conceptual, considerando variaciones lingüísticas de Brasil, y establecimiento de la versión final. Los especialistas de las diferentes etapas eran profesionales con experiencia académica o en salud pública. Para las evaluaciones de las etapas 5 y 6 se utilizó el índice de validez de contenido, considerándose como aceptable una concordancia de al menos 80% entre los especialistas.

**Resultados:**

Las 20 preguntas de la escala y sus ítems adicionales (un total de 74 ítems a responder) fueron traducidos y adaptados según las necesidades identificadas en las diferentes etapas del proceso. En las etapas 5 y 6, las preguntas de los tres dominios de la escala (evidencia, contexto y facilitación) presentaron adecuada validez de contenido. Dos preguntas presentaron un índice de validez de contenido <80% en alguna de las equivalencias. Esos y otros ítems con sugerencias de modificaciones fueron ajustados, generándose la versión final de la escala.

**Conclusión:**

La escala fue traducida y adaptada transculturalmente para el contexto brasileño, presentando adecuada validez de contenido.

**Ethical aspects**

This research respected ethical principles, having obtained the following approval data:

Research ethics committee Universidade Federal de Santa Catarina

Opinion number: 6,587,166

Approval date: 18/12/2023

Certificate of submission for ethical appraisal: Attached.

Informed consent record: Obtained from all participants prior to data collection.

## Introduction 

Promoting health and proposing changes to services are fundamental to improving clinical outcomes and delivering better quality care ^1^ ^,^ ^2^. These changes are more likely to succeed when they are based on solid evidence and take place within a supportive organizational context ^1^ ^,^ ^3^. The organizational context is essential for adopting and sustaining new initiatives or changing existing ones, as it provides the conditions needed to implement them. Thus, managers, health professionals, and local leaders must assess in advance the contextual factors that may influence the implementation of these actions. Assessing before implementing allows those involved to be aware, prepared, and committed to the change process ^4^.

The Organizational Readiness to Change Assessment (ORCA) scale, developed in the United States in 2009, has been utilized in various contexts to evaluate organizational readiness for change in healthcare services ^5^ ^,^ ^6^. It is based on the Promoting Action on Research Implementation in Health Services framework, published in 1998, which seeks to understand why specific actions are successful or not ^7^. This framework examines the complexity of implementation through contextual analysis ^8^, acknowledging that the process is intertwined with the organization as a whole, rather than just the individual. Moreover, it assesses three interactive domains: evidence, context, and facilitation (2,5,6).

The ORCA scale considers the quality of the intervention evidence, the organizational context’s capacity to support change, and the facilitation resources available for its implementation ^5^. Therefore, its use is relevant, since the impact on public health depends on the readiness of health teams to adopt new actions.

Despite ORCA’s potential to assess the implementation of actions within Brazilian health services, no studies were found that address its cultural adaptation to the local reality. Such adaptation is essential to assess the responsiveness of organizations, whether public or private, to the initiatives implemented. These initiatives often fail due to the lack of organizational readiness of leaders and teams ^9^. It is necessary to understand the perspective of professionals and managers regarding their preparedness for change ^2^. This process may favor the dissemination and effective use of the scale, which is essential for adopting and sustaining actions. Therefore, this study aims to translate and cross-culturally adapt the ORCA scale to the Brazilian context.

## Methods 

This study focuses on the translation and cross-cultural adaptation of the ORCA scale to the Brazilian context. 

The Organizational Readiness to Change Assessment scale

The ORCA scale is used to assess an organization’s readiness to promote changes in health services and to implement interventions ^5^ ^,^ ^6^. In the context of this cross-cultural adaptation, interventions are understood as projects, actions, practices, strategies, or action proposals, developed based on the identification of problems, needs, and determinants. They may also be defined as sets of preventive activities, designed with well-defined objectives and implemented within a given time frame and context, supported by a clear theoretical and methodological framework. 

The scale has 20 questions: five related to evidence, six to context, and nine to facilitation. In 18 of these questions, there are additional items ranging from three to five, totaling 74 items to be answered. Responses are provided using a five-point Likert scale, where “1 = strongly disagree” and “5 = strongly agree”, plus an option “99 = don’t know/not applicable”. In two items, the response options are “1 = very weak”, “2 = weak”, “3 = moderate”, “4 = strong”, “5 = very strong”, and “99 = don’t know/not applicable”.

The scale does not propose cut-off points to classify the level of readiness for change. However, the final score can be calculated by summing the points from the responses collected at the beginning (for planning), during (if feasible and necessary for adjustments), and after the implementation or intervention (six months or one year later). Differences in scores can be analyzed across different stages of the intervention. Additionally, the outcomes for each site and the implementation process of the interventions can be examined individually or comparatively. The total score may range from 74 to 370 points (1-5 points per item). The higher the score, the greater the readiness of the health service for change.

The scale assesses evidence, context, and facilitation ^5^. Regarding the quality of evidence for the proposed intervention, sources may include scientific research or participation in formal experiments; evidence from clinical experience or professional knowledge; evidence from user preferences or experiences, including those of caregivers and family members; routine information derived from the local practice context, which differs from professional experience as it pertains to the collective environment rather than the individual.

Regarding the quality of the organizational context to support practice change, aspects such as organizational environment, culture, leadership style, and available resources are considered. Regarding organizational capacity for facilitation, the focus is on the team’s ability to implement the intervention and provide necessary support. 

Translation and cross-cultural adaptation process

**Figure 1:**
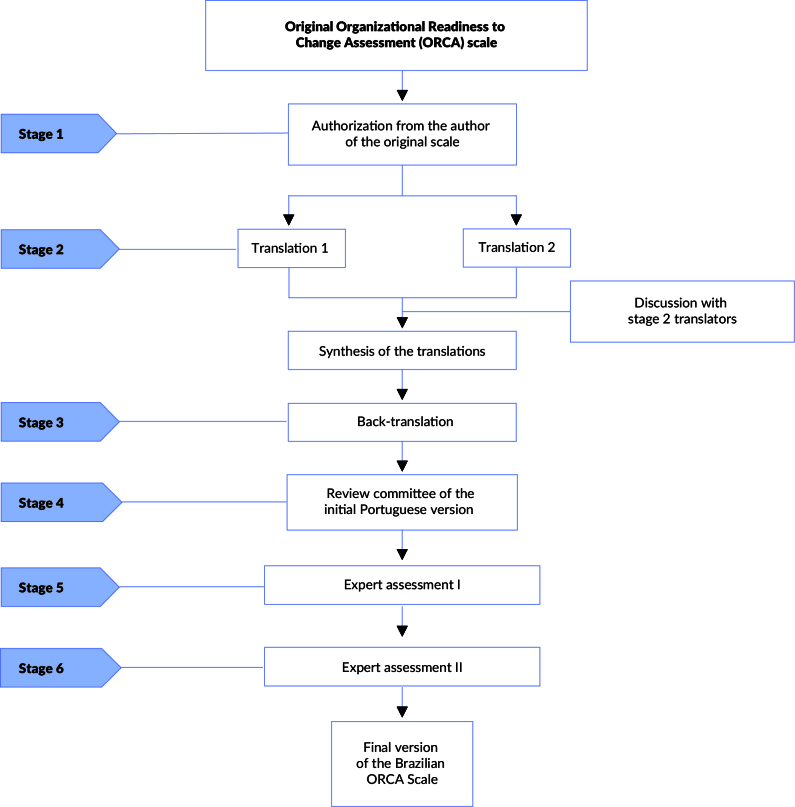
Stages of translation and cross-cultural adaptation of the Organizational Readiness to Change Assessment (ORCA) scale. Brazil, 2024

The process of translation and cross-cultural adaptation to the Brazilian context followed the recommendations of the Consensus-based Standards for the Selection of Health Measurement Instruments and previous studies published on the subject ^10^ ^,^ ^11^, and was carried out in six stages (Figure 1): 1) authorization from the author of the original scale (US English); 2) translation from English to Portuguese by two native English speakers fluent in Portuguese and synthesis of the translations into a single version; 3) back-translation of the Portuguese version into English by a native Portuguese speaker fluent in English; 4) review committee of the initial Portuguese version: clarity, comprehension, and suitability to the Brazilian context; 5) expert assessment I: semantic, idiomatic, cultural, and conceptual equivalences; 6) expert assessment II consideration of regional linguistic variations and establishment of the final version of the scale.

*Stage 1. Authorization from the author of the original scale (US English)*

Contact was made via e-mail with the lead and corresponding author of the original scale to request authorization for its translation into Portuguese and for cross-cultural adaptation to the Brazilian context. The author reviewed and approved the methodology proposed for this study and consented to the use of the instrument.

*Stage 2. Translation of the scale from English into Portuguese*

The original scale was translated by two independent translators, native to countries where English is the official language and fluent in Portuguese. Both generated versions were stored in a Microsoft Excel spreadsheet, compared by three researchers responsible for this study, and subsequently synthesized into a single version. Items that raised doubts during the synthesis were discussed among the researchers and translators to obtain the version for back-translation. 

*Stage 3. Back-translation of the Portuguese version into English*

The synthesized translation obtained after stage 2 was back-translated into English by one Brazilian translator fluent in English. The back-translation was compared with the original version of the scale to identify possible semantic and conceptual discrepancies and to adjust and adapt the Portuguese version for use in subsequent stages of assessment.

*Stage 4. Review committee of the initial Portuguese version*

The initial Portuguese version of the scale, obtained after stage 3, was evaluated by a committee of experts with professional experience in different areas and positions in the context of public health in Brazil. The members of this committee were selected through convenience sampling and identified based on their professional or academic interactions with one of the authors of this study. This committee was composed of ten experts: one university professor with knowledge and experience in public health research, one postdoctoral student, three doctoral students, one master’s student, one public health resident, and three professionals working in primary health care units of the Brazilian National Health System (SUS).

These specialists were contacted by e-mail, invited to join the committee, and informed about the objectives and importance of the activity. Upon acceptance, they were sent the Informed Consent Form for review and signature, along with a preliminary version of the scale and a glossary. The authors of this study prepared a glossary to elucidate concepts used by the scale in the Brazilian context. A face-to-face meeting was then held to discuss the scale and the glossary, addressing, in general, the clarity and comprehensibility of the items and their suitability for the Brazilian context, particularly in relation to public health. The meeting was recorded and lasted three hours. The committee members’ suggestions were recorded in a specific spreadsheet, analyzed, and used to modify certain items of the scale and the glossary. 

*Stage 5. Assessment of experts I*

After implementing the changes to the scale in the previous stage, the resulting version was emailed to the same group of specialists who participated in stage 4. They were asked to evaluate the following equivalences: semantic (meaning of words in relation to vocabulary and grammar), idiomatic (meaning of expressions), cultural (contextual appropriateness within Brazilian culture), and conceptual (preservation of the original scale’s concepts) ^12^. 

The experts assessed each item of the scale individually as extremely adequate, adequate, or inadequate. For items considered inadequate, they were asked to indicate the problem observed and to provide a suggestion for rewriting the item. The results of the assessments were tabulated, discussed, and analyzed, and pertinent adjustments were made in accordance with the experts’ suggestions. 

*Stage 6. Assessment of experts II*

In stage 6, the version of the scale obtained after stage 5 was evaluated by a second group of public health specialists. This group included representatives from the five Brazilian regions, with one expert from each region and two experts from the Northern region. Additionally, one participant had prior experience working at the Brazilian Ministry of Health. Therefore, seven experts, also selected by convenience sampling, participated in stage 6. They were identified based on professional or academic interactions with one or more of the authors of this study. The objective of this stage was to ensure the comprehension of the terms used in the scale, considering linguistic variations across the country. Efforts were made to select words with the same meaning in all regions of Brazil. After the professionals agreed to participate, the Informed Consent Form was sent for signature, along with the scale and glossary for prior review and analysis before the consensus meetings.

Two consensus meetings were held via the Zoom platform. The first lasted three hours, and the second lasted one and a half hours. The suggestions made by the experts were recorded in a Microsoft Excel spreadsheet, analyzed, and discussed, and the scale was modified as needed. After these adjustments, the scale was sent to the same group of participants in this stage, along with the glossary, for assessment of semantic, idiomatic, cultural, and conceptual equivalence. The results of the assessment were tabulated, discussed, and analyzed, and the necessary adjustments were made. This process resulted in the final version of the scale, which is available upon request to the corresponding author of this study.

Data analysis

For assessing individual agreement on the items, the content validity index was used to analyze the experts’ evaluations of semantic, idiomatic, cultural, and conceptual equivalence. 

Agreement among the experts was considered to have been reached when at least 80.0% (+) of the assessments rated the item as adequate or extremely adequate. Items with agreement values below 80.0% (--) were revised based on the experts’ suggestions. The glossary definitions and operational procedures for applying the scale were also modified by consensus among the researchers. 

## Results

The ORCA scale was translated into Portuguese and adapted to the Brazilian context. It was then evaluated by two groups of experts regarding semantic, idiomatic, cultural, and conceptual equivalences. After analyzing the different stages, we present the main results observed during the translation and adaptation process of the scale.

The first group of experts proposed suggestions for changes, primarily related to linguistic characteristics, which the researchers incorporated into the second version. They reported some difficulties in interpreting and understanding the scale, suggesting modifications to improve clarity, comprehension, and objectivity. 

In stage 5, the first group of experts evaluated the scale in terms of semantic, idiomatic, cultural, and conceptual equivalences. There was 100.0% (++) agreement on all equivalences for the evidence items, which were considered adequate or extremely adequate. Regarding the context items, three were rated above 80.0% (+) in one or more equivalences, and for facilitation, four items scored above 80.0% (+) in at least one equivalence. 

The suggestions were considered by the researchers, resulting in changes to some items (the main modifications are presented in a document available upon request from the corresponding author of this study). These changes generated a new version of the scale. The data regarding the evaluation of this stage are presented in Table 1.

In stage 6, the same steps outlined in stage 5 were carried out with a second group of expert representatives from all regions of Brazil, including one representative who had previously worked at the Brazilian Ministry of Health. The aim was to verify linguistic characteristics and perform regional cultural adaptations. 

In the evaluation by expert group II, regarding semantic, idiomatic, cultural, and conceptual equivalences, three items (the question or one of its alternatives) related to evidence received scores above 80.0% (+). As for the context items, three of them (in the item, or in one of its alternatives) were evaluated with 80.0% or more (+) in one or more equivalences, and one of them was evaluated with 80.0% or less (--) in one of the equivalences. In facilitation, five items (in the item, or in one of its alternatives) received 80.0% or more (+) in one or more equivalences, and one item received 80.0% or less (--) in three equivalences. 

A summary of the evaluation results is presented in Table 1. The suggestions were analyzed and discussed, and the necessary adjustments were made for the final version of the scale. 

The main difficulties and discrepancies encountered during the translation procedure were associated with words/terms or their specific meanings in Portuguese, considering the Brazilian context, resulting in 428 minor adjustments. After the evaluation, the researchers modified the sentences related to language characteristics to make them more understandable and more objective in Brazilian Portuguese. Thus, almost all items underwent minor adjustments.

The glossary was developed to clarify certain concepts highlighted in the evaluation by both Expert Group I and Expert Group II. Presenting a concept for the domains of evidence, context, and facilitation was necessary to achieve a better understanding of the scale. The glossary is available upon request from the corresponding author of this study.

The most significant discrepancies in the evaluations of semantic, idiomatic, cultural, and conceptual equivalence occurred among experts in group II (professionals from different regions of Brazil and the one with previous experience at the Brazilian Ministry of Health) (Table 1). In the evidence domain, all items scored above 80.0% (+). In the context domain, item 10, regarding idiomatic equivalence, was rated below this value (-67.0%) (--) by expert group II. In the facilitation domain, question 14 was rated below this value by expert group II with respect to semantic, cultural, and idiomatic equivalences. These items were discussed with the research team, and some suggestions from expert group II were adopted to clarify and simplify the evaluated items. For better understanding, the final version of the scale is available upon request from the corresponding author of this study. 

## Discussion

The results of this study show that the version of the scale translated and adapted to the Brazilian context, which assesses Organizational Readiness for Change in Health Services, presented adequate content validity regarding semantic, idiomatic, cultural, and conceptual equivalences. Semantic adjustments and modifications were necessary, as 16 of the 20 items on the scale were modified, resulting in a total of 428 adjustments. Most of these adjustments were related to words and expressions specific to Brazilian Portuguese in the health context. These adjustments aimed to make the sentences clearer and more easily understood.

The construction of a glossary with definitions of terms and sentences may contribute to a better understanding of the scale, as previously identified in the Organizational Readiness for Change ^13^ scale. Initially, the definitions referred to the domains of the Promoting Action on Research Implementation in Health Services framework (evidence, context, and facilitation), which are used and assessed by the scale, followed by adjustments in the items and alternatives, taking into account cultural and contextual aspects of the Brazilian Portuguese language and culture.

In the evidence domain, discussions focused on the type of evidence considered by the scale. In the context of Brazilian public health, adjustments were made to words with different meanings; for example, “recompensam” was replaced with “reconhecem”. In facilitation, specific changes were implemented, including the adaptation of the expression “campeão clínico” to “responsável” or “profissional líder/coordenador,” depending on the context, among other adjustments to Brazilian culture.

**Table 1:** Content^a^ validity of the Brazilian Portuguese version of the Organizational Readiness to Change Assessment (ORCA) scale based on the evaluation by Expert Groups I and II. Brazil, 2024

Domain	Expert assessment I			Expert assessment II			
	Semantics	Idiomatic	Cultural	Conceptual	Semantics	Idiomatic	Cultural	Conceptual
General								
Title	++^b^	++	++	++	++	+^c^	++	++
Scale	++	++	++	++	++	++	++	++
Enunciate	++	++	++	++	++	++	++	++
Evidence								
i1	++	++	++	++	++	++	++	++
i2	++	++	++	++	++	++	++	++
i3	++	++	++	++	++	+	++	++
i4	++	++	++	++	++	+	++	+
i5	++	++	++	++	+	+	++	++
Context								
i6	+	++	+	++	++	++	++	++
i7	++	++	++	++	++	++	++	++
i8	+	++	+	++	++	+	++	++
i9	++	++	+	++	+	+	++	++
i10	++	++	++	++	+	--^d^	+	+
i11	++	++	++	++	++	++	++	++
Facilitation								
i12	++	++	++	++	+	+	+	++
i13	++	++	+	++	+	+	++	++
i14	+	++	+	++	--	--	+	--
i15	++	++	++	++	++	++	++	++
i16	++	++	++	++	++	++	++	++
i17	++	++	++	++	++	++	+	++
i18	+	++	+	++	++	++	++	++
i19	++	++	++	++	++	++	++	++
i20	+	++	++	++	+	++	++	++

The domain of scientific and technical evidence is essential for building more effective, safe, and sustainable health policies and actions. By basing decisions on concrete data and updated studies, managers and health professionals can accurately identify the needs of the population, prioritize high-impact interventions, and avoid resource waste. In addition, the use of evidence enables continuous evaluation of results, favoring adjustments and improvements in implemented processes ^14^.

The context domain allows interventions to be tailored to local realities, strengthening decision-making, community engagement, and equity ^8^ ^,^ ^15^. This domain considers factors such as culture, leadership, and evaluation, which are essential for implementation, as demonstrated in hospitals in China ^15^.

In the facilitation domain, facilitators are recognized for their skills and lead the implementation processes. A situational diagnosis helps to understand the relationships among the elements involved in facilitating an intervention ^3^ ^,^ ^16^.

Instruments similar to the ORCA scale aim to measure a team’s preparedness to implement new actions. In 2025, a systematic review of the ORC (similar to the ORCA) investigated implementation mediators and moderators. Forty-seven studies were included, focusing on the psychological and behavioral preparedness of organizational members. The review highlighted the lack of conceptual clarity and recommended glossaries to support interpretations ^13^, reinforcing the importance of the resource developed in this study.

The Brazilian National Health System (SUS) provides free, comprehensive, and universal healthcare, making it one of the largest public health systems in the world ^17^. The translation of the scale sought to ensure alignment with the reality of SUS. The entire translation and adaptation process, including the selection of experts and modifications to the items, was carried out with this focus. However, the scale can also be applied in other contexts, such as the supplementary health sector. The goal is to ensure that the scale is culturally sensitive and linguistically appropriate in the new language.

This study has limitations. The psychometric properties of the scale have not yet been tested. Moreover, no cut-off point has been defined to indicate whether an organization is ready for change. Analyses should consider comparisons between studies and organizations, as well as the use of focus groups or interviews to investigate local barriers and facilitators.

The Brazilian Portuguese version of the ORCA is brief (takes about 20 minutes to complete), easy to administer, and presents good content validity. Its use may support the assessment of organizational readiness for change in health services that aim to identify the degree of propensity for change among health professionals in Brazil. Its application favors the adoption, implementation, and sustainability of actions in health services and systems, potentially generating a greater impact on public health. 

It is recommended that future studies test the psychometric properties of the scale and its validity in different contexts. The findings indicate that the Portuguese version is suitable for application.
