# Supplementary material for: Translation and cultural adaptation to the Brazilian context of the Organizational Readiness to Change Assessment scale
Source: Epidemiol Serv Saude. 2026 Mar 9;35:e202400921. doi: 10.1590/S2237-96222025v34e20240921.en (PMC12991398; doi:10.1590/S2237-96222025v34e20240921.en)
Supplement: Translation

# Translation: Tradução e adaptação ao contexto brasileiro da escala Organizational Readiness to Change Assessment

**DOI:** 10.1590/S2237-96222025v34e20240921.pt

**Authors:** Tânia R. Bertoldo Benedetti, Thaís Fávero Alves, Natalia Santos, Cassiano Ricardo Rech, Gaia Salvador Claumann, Fabiana Brito Silva

**Affiliations:** 1 Universidade Federal de Santa Catarina, Departamento de Educação Física, Florianópolis, SC, Brasil; 2 University of Nebraska Medical Center, College of Public Health, Omaha, NE, Estados Unidos

**Keywords:** Avaliação de Programas e Instrumentos de Pesquisa, Comparação Transcultural, Mecanismos de Avaliação da Assistência à Saúde, Avaliação de Serviços de Saúde, Estudos de Validação.

## Abstract

**Objetivos:**

Traduzir e adaptar transculturalmente, para o contexto brasileiro, a escala Organizational Readiness to Change Assessment*.*

**Métodos:**

O processo de tradução e de adaptação transcultural ocorreu em seis etapas: 1) anuência do autor da escala original; 2) duas traduções da escala para o português e síntese em uma versão; 3) retrotradução; 4) comitê de avaliação da versão inicial (dez participantes): clareza, compreensão e adequação ao contexto brasileiro; 5) avaliação de especialistas I (mesmos da etapa anterior): avaliação das equivalências semântica, idiomática, cultural e conceitual; 6) avaliação de especialistas II (sete especialistas: cinco regiões do Brasil): avaliação das equivalências semântica, idiomática, cultural e conceitual, considerando variações linguísticas do Brasil, e estabelecimento da versão final. Os especialistas das diferentes etapas eram profissionais com experiência acadêmica ou profissionais da saúde pública. Para as avaliações das etapas 5 e 6, utilizou-se o índice de validade de conteúdo, considerando-se como aceitável uma concordância de pelo menos 80,0% entre os especialistas.

**Resultados:**

As 20 questões da escala e seus itens adicionais (totalizando 74 itens para serem respondidos) foram traduzidos e adaptados conforme necessidades identificadas nas diferentes etapas do processo. Nas etapas 5 e 6, as questões dos três domínios da escala (evidência, contexto e facilitação) apresentaram adequada validade de conteúdo. Duas questões apresentaram índice de validade de conteúdo <80,0% em alguma das equivalências. Esses e outros itens com sugestões de alterações foram ajustados, gerando-se a versão final da escala.

**Conclusão:**

A escala foi traduzida e adaptada transculturalmente para o contexto brasileiro, apresentando adequada validade de conteúdo.

**Aspectos éticos**

Esta pesquisa respeitou os princípios éticos, obtendo os seguintes dados de aprovação:

Comitê de ética em pesquisa: Universidade Federal de Santa Catarina

Número do parecer: 6.587.166

Data de aprovação: 18 de dezembro de 2023

Certificado de apresentação de apreciação ética: Em anexo.

Registro do consentimento livre e esclarecido: Obtido de todos os participantes antes da coleta.

## Introdução 

A promoção da saúde e a proposição de mudanças nos serviços são fundamentais para melhorar os resultados clínicos e a qualidade do atendimento ^1^ ^,^ ^2^. Essas mudanças têm mais chances de sucesso quando baseadas em evidências consolidadas e em um contexto organizacional favorável ^1^ ^,^ ^3^. O contexto organizacional é essencial para a adoção e a sustentabilidade de novas ações ou alterações nas já existentes, oferecendo condições para a sua realização. Assim, é importante que gestores, profissionais da saúde e lideranças locais analisem previamente os fatores contextuais que possam impactar na implementação das ações. Avaliar antes de implementar permite que os envolvidos estejam cientes, preparados e comprometidos com o processo de mudança ^4^.

A escala Organizational Readiness to Change Assessment (ORCA), criada em 2009 nos Estados Unidos, tem sido utilizada em diferentes contextos para avaliar a prontidão organizacional para mudanças em serviços de saúde ^5^ ^,^ ^6^. Ela se baseia na ferramenta Promoting Action on Research Implementation in Health Services, publicada em 1998, que busca compreender por que determinadas ações são ou não bem-sucedidas ^7^. Essa ferramenta explora a complexidade da implementação por meio da análise do contexto ^8^, reconhecendo que o processo está relacionado à organização como um todo, não apenas ao indivíduo, e avalia três domínios interativos: evidência, contexto e facilitação (2,5,6).

A ORCA considera a qualidade das evidências da intervenção, do contexto organizacional para apoiar a mudança e da facilitação, relacionada à capacidade organizacional de implementá-la ^5^. Assim, torna-se relevante sua utilização, já que o impacto na saúde pública depende da prontidão da equipe de saúde para adotar novas ações.

Apesar do potencial da escala ORCA para avaliar a implementação de ações nos serviços de saúde brasileiros, não foram encontrados estudos de adaptação cultural da escala à nossa realidade. Essa adaptação é essencial para avaliar a capacidade de resposta das organizações, sejam públicas ou privadas, às iniciativas implementadas. Muitas vezes, elas falham por falta de prontidão organizacional das lideranças e das equipes ^9^. É necessário compreender a perspectiva de profissionais e de gestores quanto à preparação para mudanças ^2^. Esse processo pode favorecer a disseminação e o uso efetivo da escala, essencial à adoção e à sustentabilidade das ações. Portanto, o objetivo deste estudo é traduzir e adaptar transculturalmente para o contexto brasileiro a escala ORCA*.*

## Métodos 

Tratou-se de estudo de tradução e de adaptação transcultural para o contexto brasileiro da escala ORCA. 

A escala Organizational Readiness to Change Assessment

A escala ORCA é utilizada para avaliar a prontidão da organização para promover mudanças nos serviços de saúde e para a implementação de intervenções ^5^ ^,^ ^6^. No contexto dessa adaptação transcultural, compreendem-se intervenções como projeto, ação, prática, estratégia, ou proposta de ação, construída a partir da identificação de problemas, de necessidades e de fatores determinantes. Ou, ainda, como um conjunto de atividades preventivas, elaboradas com base em objetivos definidos e implementados em um determinado tempo e contexto, com fundamentação teórica e metodológica bem definida. 

A escala possui 20 questões, sendo cinco relacionadas à evidência, seis ao contexto e nove à facilitação. Em 18 questões, há itens adicionais que variam, em quantidade, de três a cinco. Assim, ao todo, a escala compreende 74 itens que necessitam de respostas. Os itens são respondidos utilizando uma escala likert de cinco pontos, sendo “1=discordo totalmente” e “5=concordo totalmente”, acrescida de uma alternativa “99=não sei/não se aplica”. Em dois itens, as opções de resposta são “1=muito fraca”, “2=fraca”, “3=moderada”, “4=forte”, “5=muito forte” e “99=não sei/não se aplica”.

A escala não propõe pontos de corte para classificar o quanto uma organização está pronta para a mudança. Porém, a pontuação pode ser obtida somando-se os pontos das respostas no início (para planejamento), durante (se possível e necessário para ajustes) e depois da implementação ou da intervenção (seis meses ou um ano após). Pode-se analisar a diferença de pontuação entre os momentos diversos da intervenção, ou verificar os resultados de cada local e como ocorrem as intervenções, podendo ser analisados individualmente por local ou comparando-os. A pontuação poderá variar de 74 a 370 pontos (1-5 pontos em cada item). Quanto maior a pontuação da escala, maior a prontidão do serviço de saúde para a mudança.

A escala considera a evidência, o contexto e a facilitação ^5^. Quando se refere à qualidade da evidência para a intervenção proposta, as fontes podem ser: evidências de pesquisas científicas ou participação em experimentos formais; evidências de experiência clínica ou conhecimento profissional; evidências de preferências ou experiências dos usuários, incluindo as de cuidadores e de familiares; e informações rotineiras derivadas do contexto da prática local, que diferem da experiência profissional por serem do domínio do ambiente coletivo e não do indivíduo.

Quanto à qualidade do contexto organizacional para apoiar a mudança prática, verifica-se como o ambiente da organização, a sua cultura, o estilo de liderança e os recursos disponíveis podem impactar o estudo. Quanto à capacidade organizacional para a facilitação da mudança, verifica-se a capacidade da equipe para implementar a intervenção e fornecer suporte. 

Processo de tradução e adaptação transcultural

O processo de tradução e de adaptação transcultural para o contexto brasileiro seguiu as recomendações do Consensus-based Standards for the Selection of Health Measurement Instruments e de estudos prévios publicados sobre a temática ^10^ ^,^ ^11^, sendo realizado em seis etapas (Figura 1): 1) anuência do autor da escala em seu formato original (inglês americano); 2) tradução do inglês para o português por dois nativos em inglês com fluência em português e síntese das traduções em uma única versão; 3) retrotradução da versão em português para o inglês, por um nativo em português com fluência em inglês; 4) comitê de avaliação da versão inicial em português: clareza, compreensão e adequação ao contexto brasileiro; 5) avaliação dos especialistas I: equivalência semântica, idiomática, cultural e conceitual; 6) avaliação dos especialistas II: consideração das variações linguísticas regionais e estabelecimento da versão final da escala. 

*Etapa 1. Anuência do autor da escala em seu formato original (inglês americano)*

Foi realizado contato, por e-mail, com o autor principal e correspondente da escala em seu formato original para solicitar autorização para a realização da tradução para o português e da adaptação transcultural para o contexto brasileiro. O autor revisou e aprovou a metodologia proposta para este estudo e consentiu na utilização do instrumento.

*Etapa 2. Tradução da escala do inglês para o português*

A tradução da escala original foi realizada por dois tradutores independentes, nativos de países cujo idioma oficial é o inglês e fluentes em português. As duas versões geradas foram armazenadas em planilha do software Microsoft Excel e comparadas por três pesquisadores responsáveis por este estudo e, posteriormente, sintetizadas em uma única versão. Os itens que geraram dúvidas quanto à síntese foram discutidos entre os pesquisadores e os tradutores para a obtenção da versão para a retrotradução. 

**Figura 1:** Etapas de tradução e de adaptação transcultural da escala Organizational Readiness to Change Assessment (ORCA). Brasil, 2024

*Etapa 3. Retrotradução da versão em português para o inglês*

A síntese das traduções, obtida após a etapa 2, foi retrotraduzida para o inglês, por um tradutor brasileiro e fluente em inglês. A retrotradução foi comparada à versão original da escala, a fim de identificar possíveis discrepâncias semânticas e conceituais, para ajustar e adaptar a versão em português para ser avaliada nas etapas seguintes.

*Etapa 4. Comitê de avaliação da versão inicial em português*

A versão inicial da escala em português, obtida após a etapa 3, foi avaliada por um comitê de especialistas com experiência profissional em diferentes áreas e cargos no contexto da saúde pública no Brasil. Os integrantes desse comitê foram selecionados por conveniência, identificados a partir de interação profissional ou acadêmica com algum dos autores deste estudo. Participaram desse comitê dez especialistas: um professor do ensino superior com conhecimento e atuação em pesquisas na área da saúde pública, um aluno de pós-doutorado, três alunos de doutorado, um aluno de mestrado, um residente em saúde publica e três profissionais atuantes em unidades básicas de saúde do Sistema Único de Saúde (SUS).

Realizou-se contato com esses especialistas por e-mail, convidando-os a compor o comitê e explicando os objetivos e a importância dessa atividade. Após o aceite, foi enviado o registro de consentimento livre e esclarecido para leitura e assinatura, além de uma versão preliminar da escala e de um glossário para a leitura. O glossário foi construído pelos autores deste estudo para elucidar conceitos utilizados pela escala no contexto brasileiro. Em seguida, foi realizada uma reunião presencial para discussão da escala e do glossário, abordando, de modo geral, a clareza e a compreensão dos itens e a adequação ao contexto brasileiro, considerando principalmente a saúde pública. A reunião foi gravada e teve duração de três horas. As sugestões dos integrantes do comitê foram registradas em planilha específica, analisadas e, a partir disso, foram realizadas alterações em alguns itens da escala e do glossário. 

*Etapa 5. Avaliação dos especialistas I*

Após as alterações realizadas na escala na etapa anterior, a versão resultante foi enviada por e-mail para o mesmo grupo de especialistas participantes da etapa 4, para avaliação das equivalências semântica (referente ao significado das palavras em relação ao vocabulário e à gramática), idiomática (referente ao significado das expressões), cultural (referente à adequação do contexto na cultura brasileira) e conceitual (referente à manutenção dos conceitos da escala original) ^12^. 

Os especialistas avaliaram cada item da escala, individualmente, como extremamente adequado, adequado ou inadequado. Para itens considerados inadequados, solicitou-se a indicação do problema observado, acompanhada de sugestão de escrita para melhorar o item. Os resultados das avaliações foram tabulados, discutidos e analisados, e os ajustes pertinentes foram realizados conforme as sugestões dos especialistas. 

*Etapa 6. Avaliação dos especialistas II*

Na etapa 6, a versão da escala obtida após a etapa 5 foi avaliada por um segundo grupo de especialistas em saúde pública. Os participantes desse grupo eram representantes das cinco regiões do Brasil, havendo um especialista de cada região e dois especialistas da região Norte. Adicionalmente, participou dessa etapa um representante com atuação prévia no Ministério da Saúde. Assim, participaram da etapa 6 sete especialistas, também selecionados por conveniência, identificados a partir da interação profissional ou acadêmica com algum dos autores deste estudo. O objetivo da avaliação, nessa etapa, era garantir a compreensão das palavras utilizadas na escala, considerando as variações linguísticas no país. Assim, buscou-se a utilização de palavras com o mesmo significado em todas as regiões do país. Após o aceite dos profissionais, foi enviado o registro de consentimento livre e esclarecido para assinatura, bem como a escala e o glossário para leitura e análise de forma prévia às reuniões de consenso.

Foram realizadas duas reuniões de consenso via plataforma Zoom. A primeira durou três horas, e a segunda durou uma hora e meia. As sugestões realizadas pelos especialistas foram registradas em uma planilha do software Microsoft Excel, analisadas e discutidas, sendo a escala modificada conforme necessidade. Após os ajustes, a escala foi enviada ao mesmo grupo participante dessa etapa, junto com o glossário, para avaliação de equivalência semântica, idiomática, cultural e conceitual. Os resultados da avaliação foram tabulados, discutidos e analisados, sendo realizados os ajustes necessários. Assim, obteve-se a versão final da escala, a qual está disponível mediante solicitação ao autor correspondente deste estudo.

Análise dos dados

No processo de avaliação da concordância individual dos itens, foi utilizado o índice de validade de conteúdo para a análise dos especialistas quanto à equivalência semântica, idiomática, cultural e conceitual. 

Considerou-se ter havido concordância entre os especialistas quando pelo menos 80,0% (+) das avaliações foram adequadas ou extremamente adequadas. Os itens com valores de concordância menores do que 80,0% (--) foram revisados com base nas sugestões dos especialistas. Também foram modificadas as definições do glossário e as operacionais de aplicação da escala a partir do consenso entre os pesquisadores. 

## Resultados 

A escala ORCA foi traduzida e adaptada para o contexto brasileiro e foi avaliada por dois grupos de especialistas quanto às equivalências semântica, idiomática, cultural e conceitual. Após análise das diferentes etapas, apresentaremos os principais resultados observados durante o processo de tradução e de adaptação da escala.

O grupo de especialistas I trouxe sugestões de mudanças relacionadas, principalmente, às características linguísticas, que foram acatadas pelos pesquisadores. Os especialistas relataram algumas dificuldades de interpretação e de compreensão da escala, realizando sugestões para melhorar clareza, compreensão e objetividade. 

Na etapa 5, os especialistas I avaliaram a escala com relação às equivalências semântica, idiomática, cultural e conceitual. Houve 100,0% (++) de concordância, quanto a todas as equivalências, sobre os itens relacionados à evidência, sendo elas consideradas adequadas ou extremamente adequadas. Quanto aos itens do contexto, três foram avaliadas com mais de 80,0% (+) em uma ou mais equivalências. E, na facilitação, quatro itens receberam mais de 80,0% (+) em pelo menos uma das equivalências. 

As sugestões foram consideradas pelos pesquisadores e resultaram em alterações sobre determinados itens (as principais alterações estão apresentadas em documento cuja consulta pode ser solicitada ao autor correspondente deste estudo). Essas alterações geraram uma nova versão da escala. Os dados referentes à avaliação dessa etapa estão apresentados na Tabela 1.

Na etapa 6, foram realizados os mesmos passos apresentados na etapa 5 com representantes especialistas II de todas as regiões do Brasil e um representante que atuou no Ministério da Saúde, a fim de verificar as características linguísticas e realizar adaptações culturais regionais. 

Na avaliação dos especialistas II, com relação às equivalências semântica, idiomática, cultural e conceitual, três itens (no item, ou em uma de suas alternativas) relacionados à evidência receberam valores superiores a 80,0% (+). Quanto aos itens do contexto, três deles (no item, ou em uma de suas alternativas) foram avaliados com 80,0% ou mais (+) em uma ou mais equivalências, e um deles foi avaliado com 80,0% ou menos (--) em uma das equivalências. Já na facilitação, cinco itens (no item, ou em um de suas alternativas) receberam 80,0% ou mais (+) em uma ou mais equivalências, e um item recebeu 80,0% ou menos (--) em três equivalências. 

A síntese da avaliação dos resultados está apresentada na Tabela 1. As sugestões foram analisadas e discutidas, sendo realizados os ajustes necessários para a versão final da escala. 

As principais dificuldades e discrepâncias encontradas durante o procedimento de tradução estiveram associadas às palavras/termos ou aos sentidos específicos no português, considerando o contexto brasileiro, resultando em 428 pequenos ajustes. Após a avaliação, os pesquisadores modificaram as sentenças relacionadas às características de linguagem para que ficassem mais claras e objetivas na língua portuguesa do Brasil. Assim, em praticamente todos os itens houve pequenos ajustes.

O glossário foi desenvolvido para deixar mais claros determinados conceitos que foram apontados na avaliação tanto com os especialistas I quanto com os especialistas II. A apresentação de um conceito para os domínios da evidência, do contexto e da facilitação foi necessária para um melhor entendimento da escala. O glossário está disponível mediante solicitação ao autor correspondente deste estudo.

As maiores discrepâncias nas avaliações das equivalências semântica, idiomática, cultural e conceitual ocorreram entre os especialistas do grupo II (profissionais de diferentes regiões do Brasil e aquele com atuação prévia no Ministério da Saúde) (Tabela 1). Com relação à dimensão da evidência, todos os itens ficaram com valores acima de 80,0% (+). Quanto à dimensão do contexto, o item 10, com relação à equivalência idiomática, na avaliação dos especialistas II, ficou abaixo desse valor, -67,0% (--). Já na dimensão da facilitação, a questão 14, na avaliação dos especialistas II, ficou abaixo desse valor com relação às equivalências semântica, cultural e idiomática. Esses itens foram discutidos com a equipe de pesquisadores e adotou-se parte das sugestões realizadas pelos especialistas II, visando deixar os itens avaliados mais compreensíveis e claros. Para melhor entendimento, a versão final da escala está disponível mediante solicitação ao autor correspondente deste estudo. 

## Discussão 

Os resultados deste estudo mostram que a versão da escala traduzida e adaptada ao contexto brasileiro, que avalia a Prontidão Organizacional para Mudanças em Serviços de Saúde, apresentou adequada validade de conteúdo quanto às equivalências semântica, idiomática, cultural e conceitual. Foram necessários ajustes e mudanças semânticas: 16 dos 20 itens da escala sofreram alterações, totalizando 428 ajustes, a maioria referente a palavras e a expressões específicas do português no contexto da saúde brasileira. Esses ajustes buscaram tornar as sentenças mais claras e compreensíveis.

A construção de um glossário com definições de termos e sentenças poderá contribuir para melhor compreensão da escala, como já identificado na Organizational Readiness for Change ^13^. Inicialmente, as definições são sobre os domínios da ferramenta Promoting Action on Research Implementation in Health Services (evidência, contexto e facilitação), utilizados e avaliados pela escala, seguidas dos ajustes nos itens e nas alternativas, respeitando elementos culturais e contextuais na língua e na cultura brasileiras.

No domínio da evidência, discutiu-se o tipo de evidência considerado pela escala. No domínio do contexto, ajustes foram feitos em palavras com sentidos distintos na saúde pública brasileira; por exemplo, “recompensam” foi substituída por “reconhecem”. Na facilitação, houve modificações pontuais; a expressão “campeão clínico” foi adaptada para “responsável” ou “profissional líder/coordenador”, conforme o contexto. Além de outros ajustes para a cultura brasileira.

**Tabela 1:** Validade de conteúdo^a^ da versão da escala Organizational Readiness to Change Assessment (ORCA) em português com base na avaliação dos especialistas I e II. Brasil, 2024

Domínio	Avaliação especialistas I				Avaliação especialistas II			
	Semântica	Idiomática	Cultural	Conceitual	Semântica	Idiomática	Cultural	Conceitual
Geral								
Título	++^b^	++	++	++	++	+^c^	++	++
Escala	++	++	++	++	++	++	++	++
Enunciad	++	++	++	++	++	++	++	++
Evidênci								
i1	++	++	++	++	++	++	++	++
i2	++	++	++	++	++	++	++	++
i3	++	++	++	++	++	+	++	++
i4	++	++	++	++	++	+	++	+
i5	++	++	++	++	+	+	++	++
Contexto								
i6	+	++	+	++	++	++	++	++
i7	++	++	++	++	++	++	++	++
i8	+	++	+	++	++	+	++	++
i9	++	++	+	++	+	+	++	++
i10	++	++	++	++	+	--^d^	+	+
i11	++	++	++	++	++	++	++	++
Facilitação								
i12	++	++	++	++	+	+	+	++
i13	++	++	+	++	+	+	++	++
i14	+	++	+	++	--	--	+	--
i15	++	++	++	++	++	++	++	++
i16	++	++	++	++	++	++	++	++
i17	++	++	++	++	++	++	+	++
i18	+	++	+	++	++	++	++	++
i19	++	++	++	++	++	++	++	++
i20	+	++	++	++	+	++	++	++

O domínio de evidências científicas e técnicas é essencial para a construção de políticas e de ações de saúde mais eficazes, seguras e sustentáveis. Ao basear decisões em dados concretos e estudos atualizados, gestores e profissionais da saúde conseguem identificar com precisão as necessidades da população, priorizar intervenções de maior impacto e evitar desperdícios de recursos. Além disso, a utilização de evidências permite a avaliação contínua dos resultados, favorecendo ajustes e melhorias nos processos implementados ^14^.

Já o domínio do contexto permite que intervenções sejam adequadas às realidades locais, fortalecendo as decisões, o engajamento comunitário e a equidade ^8^ ^,^ ^15^. Esse domínio considera fatores como cultura, liderança e avaliação, fundamentais para a implementação, o que já foi demonstrado em hospitais na China ^15^.

No domínio da facilitação, os facilitadores são reconhecidos por suas habilidades e lideram os processos de implementação. Um diagnóstico situacional contribui para entender as relações entre os elementos da facilitação de uma intervenção ^3^ ^,^ ^16^.

Instrumentos semelhantes à escala ORCA buscam medir o preparo das equipes para implementar novas ações. Em 2025, uma revisão sistemática sobre a ORC (semelhante à ORCA) investigou mediadores e moderadores de implementação. Foram incluídos 47 estudos, com foco na preparação psicológica e comportamental dos membros da organização. A revisão destacou a falta de clareza conceitual e recomendou glossários para apoiar interpretações ^13^, reforçando a importância do recurso desenvolvido neste estudo.

No Brasil, o SUS oferece atendimento gratuito, integral e universal, e é considerado um dos maiores sistemas públicos de saúde do mundo ^17^. A tradução da escala buscou adequação à realidade do SUS. Todo o processo de tradução e de adaptação, incluindo a seleção de especialistas e as alterações nos itens, teve esse foco. No entanto, a escala também pode ser aplicada em outros contextos, como a saúde suplementar. O objetivo é garantir que a escala seja culturalmente sensível e linguisticamente adequada na nova língua.

Este estudo apresenta limitações. As propriedades psicométricas da escala ainda não foram testadas. Também não há ponto de corte definido para indicar se a organização está pronta para a mudança. A análise deve considerar comparações entre estudos e organizações, além de utilizar grupos focais ou entrevistas para investigar barreiras e facilitadores locais.

A versão em português da ORCA é curta (leva cerca de 20 minutos para preenchimento), de fácil aplicação e apresenta boa validade de conteúdo. Seu uso pode apoiar a avaliação da prontidão organizacional para mudanças em serviços de saúde que se proponham a identificar o nível de propensão à mudança dos profissionais de saúde no Brasil. A sua utilização favorece a adoção, a implementação e a sustentabilidade de ações nos serviços e nos sistemas de saúde, podendo gerar um maior impacto na saúde pública. 

Recomenda-se que estudos futuros testem as propriedades psicométricas da escala e a sua validade em diferentes contextos. Os achados indicam que a versão em português está adequada para aplicação
